# Supplementary material for: Phage resistance profiling identifies new genes required for biogenesis and modification of the corynebacterial cell envelope
Source: eLife. 2022 Nov 9;11:e79981. doi: 10.7554/eLife.79981 (PMC9671496; doi:10.7554/eLife.79981)
Supplement: Figure 1—source data 1. — This table contains the whole-genome sequencing data for the lab-adapted phages. [file elife-79981-fig1-data1.docx]

**Figure 1—source data 1. SNPs detected in phages adapted to MB001.**

| Phage isolate | Sequencing method | Nucleotide | Amino Acid change | Gene product | Predicated function |
| --- | --- | --- | --- | --- | --- |
| ^WT^CL31* | WGS | T19334G | Leu68Val | *clg_38* | hypothetical protein |
| ^CP^CL31.a | WGS | T19334G | Leu68Val | *clg_38* | hypothetical protein |
|  |  | A24020G | Met1088Thr | *clg_55* | minor tail protein / capsid maturation protease |
|  |  | A24020G | Silent | *clg_56* | tail fiber protein |
| ^CP^CL31.b* | Sanger, WGS | T19334G | Leu68Val | *clg_38* | hypothetical protein |
|  |  | T24296A | Asp996Val | *clg_55* | minor tail protein / capsid maturation protease |
| ^CP^CL31.c | Sanger | T24030C | Met1088Val | *clg_55* | minor tail protein / capsid maturation protease |
|  |  | T24030C | Asp2Gly | *clg_56* | tail fiber protein |
| ^CP^CL31.d | Sanger | T24030C | Met1088Val | *clg_55* | minor tail protein / capsid maturation protease |
|  |  | T24030C | Asp2Gly | *clg_56* | tail fiber protein |
| Cog* | WGS | A19202T | Asp94Val | *gp21* | Minor tail protein |

*indicates the isolates used throughout the report
