# Supplementary material for: Phage resistance profiling identifies new genes required for biogenesis and modification of the corynebacterial cell envelope
Source: eLife. 2022 Nov 9;11:e79981. doi: 10.7554/eLife.79981 (PMC9671496; doi:10.7554/eLife.79981)
Supplement: Figure 2—source data 1. — This table contains the metadata associated with the Tn-Seq runs. [file elife-79981-fig2-data1.docx]

**Figure 2—source data 2. Phage challenge tn-seq metadata**

| Sample | Replicate | CFU collected | Number of reads | Number of unique insertions |
| --- | --- | --- | --- | --- |
| No phage | 1 | 2.64E+06 | 11,649,827 | 132,392 |
| No phage | 2 | 4.10E+06 | 12,848,601 | 140,071 |
| Cog | 1 | 2.20E+05 | 94,253 | 7,434 |
| Cog | 2 | 3.29E+05 | 82,470 | 1,584 |
| WT-CL31 | 1 | 6.41E+05 | 67,180 | 1,799 |
| WT-CL31 | 2 | 6.60E+05 | 62,604 | 1,752 |
| CP-CL31 | 1 | 7.22E+04 | 78,246 | 2,650 |
| CP-CL31 | 2 | 1.60E+05 | 62,355 | 1,365 |
