# Supplementary material for: Phage resistance profiling identifies new genes required for biogenesis and modification of the corynebacterial cell envelope
Source: eLife. 2022 Nov 9;11:e79981. doi: 10.7554/eLife.79981 (PMC9671496; doi:10.7554/eLife.79981)
Supplement: Figure 7—source data 1. — This table contains the whole-genome sequencing results from MB001 survivors following WTCL31 challenged. [file elife-79981-fig7-data1.docx]

**Figure 7—table supplement 1. SNPs in *Cglu* following challenge with ^WT^CL31.**

| Isolate | Gene product | Nucleotide | Amino Acid change |
| --- | --- | --- | --- |
| ACM91 | *pccB* | C2832292G | Met163Ile |
| ACM93 | *cgp_0475* | 419547+GAGGTCCTCCCACTC | +EVLPL |
| ACM94 | *cgp_0475* | 419547+GAGGTCCTCCCACTC | +EVLPL |
| ACM95 | *otsA* | G2563380C | Gly219Arg |
| ACM97 | noncoding intergenic | G2766762A |  |
| ACM97 | *deoC* | C405770T | Gln111* |
| ACM98 | *fadD2* | C2839091T | Met255Ile |
| ACM100 | *pccB* | C2831380A | Met467Ile |
| ACM103 | *cgp_0579* | G520949T | Arg42Leu |
| ACM105 | *cgp_1758* | C1632854T | Silent |
| ACM105 | noncoding upstream *cgp_0475* | G419718T |  |
| ACM106 | *fadD2* | C2839091T | Met255Ile |
