# Supplementary figures and images for: Phage resistance profiling identifies new genes required for biogenesis and modification of the corynebacterial cell envelope
Source: eLife. 2022 Nov 9;11:e79981. doi: 10.7554/eLife.79981 (PMC9671496; doi:10.7554/eLife.79981)

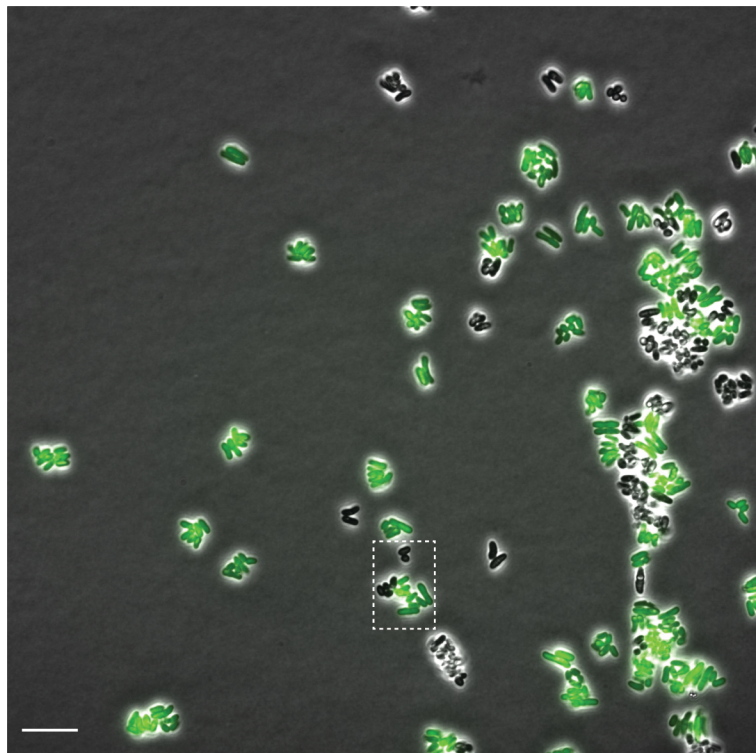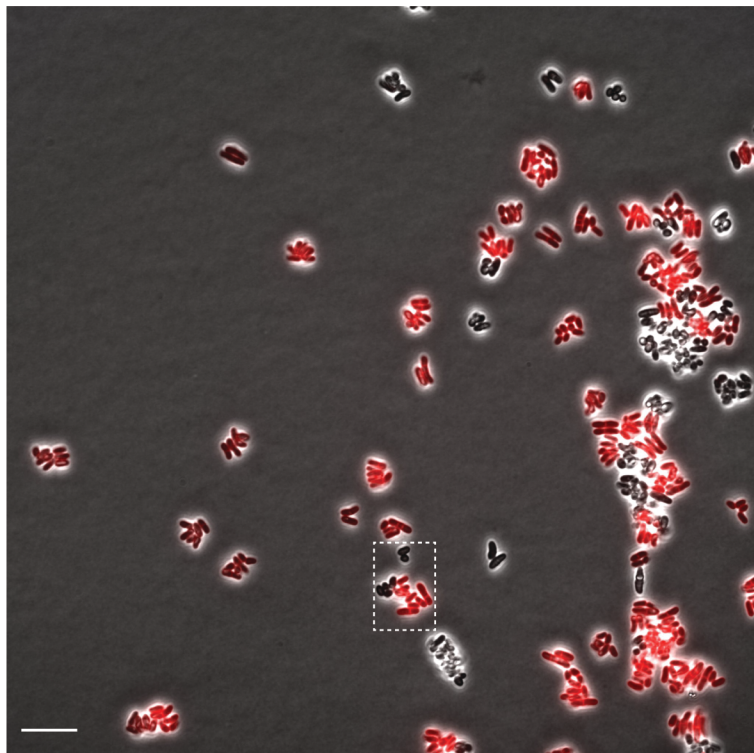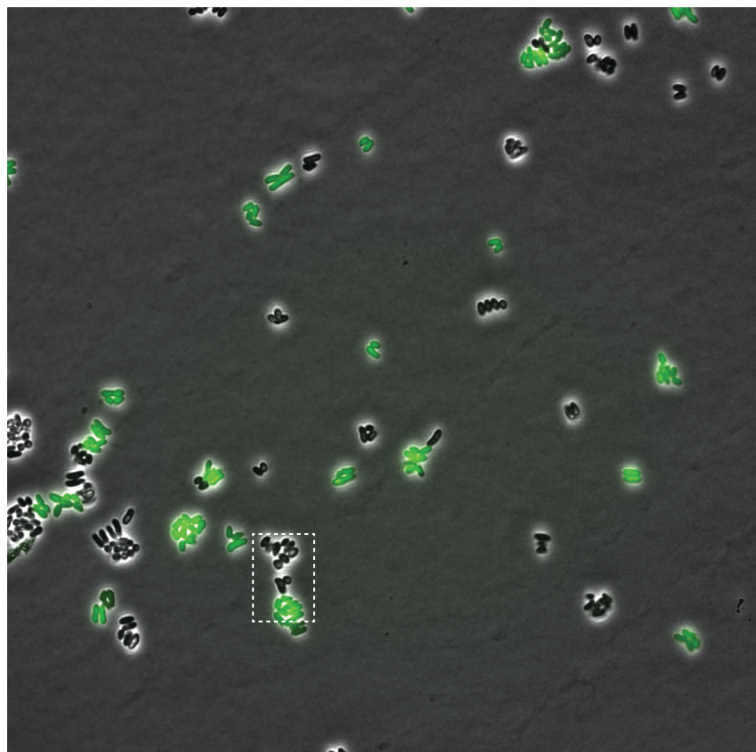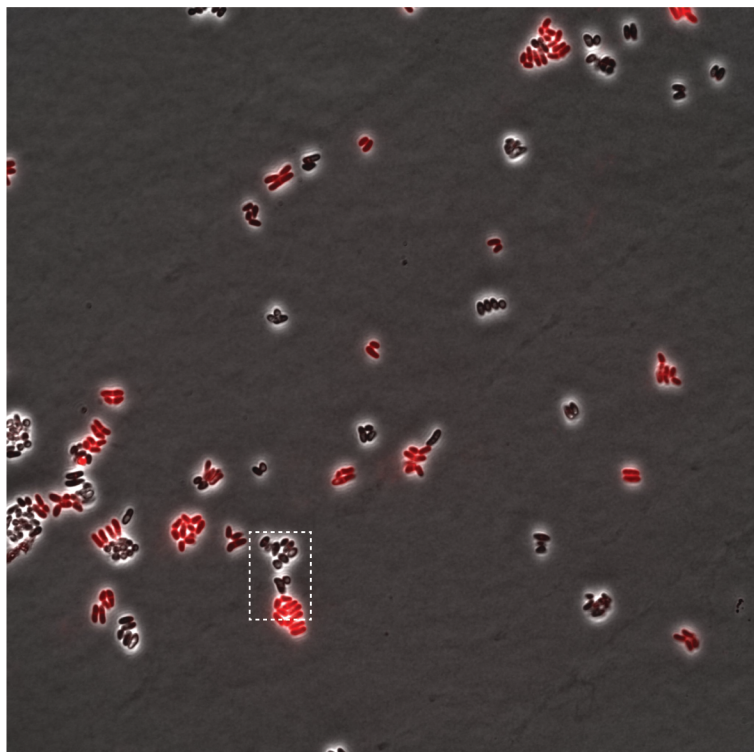

Supplement: Figure 8—source data 1. — Wild-type (WT) cells expressing gfp or unlabeled Δpks (left) or ΔahfA (right) cells lacking a fluorescent protein marker were grown to an OD600 between 0.15 and 0.4 depending on strain fitness, stained with 6-TMR-Tre for 30 minutes, washed, mixed together, and then applied to an agarose pad for visualization by fluorescence microscopy. Scale bar represents 10 µm and white box indicates area cropped for enlarged figure. [file elife-79981-fig8-data1.pdf]

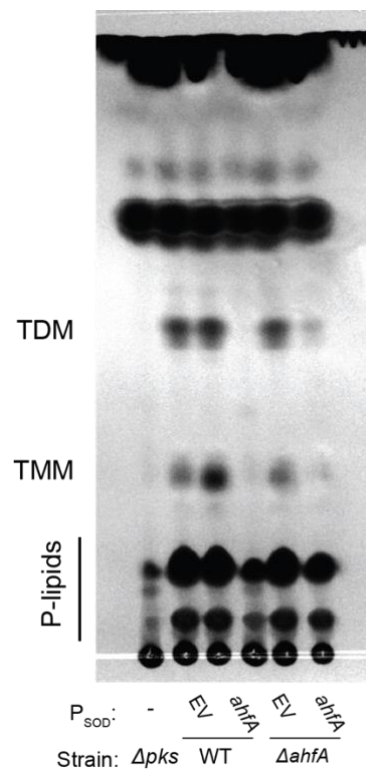

Supplement: Figure 8—source data 3. — The plate was developed in chloroform:methanol:water (30:8:1), dipped in primuline, and spots visualized with UV. Key species are labeled: TMM, trehalose monomycolate; TDM, trehalose dimycolate; P-lipids, phospholipids. The strains in panels B and C contained an empty vector (EV) or the indicated gene constitutively expressed from the PSOD promoter. [file elife-79981-fig8-data3.pdf]
