# Supplementary material for: Phage resistance profiling identifies new genes required for biogenesis and modification of the corynebacterial cell envelope
Source: eLife. 2022 Nov 9;11:e79981. doi: 10.7554/eLife.79981 (PMC9671496; doi:10.7554/eLife.79981)
Supplement: Supplementary file 1. [file elife-79981-supp1.docx]

**Supplementary File 1. Primers used in this study.**

| Name | Sequence (5'-3') | Function | Ref. |
| --- | --- | --- | --- |
| ACM96 | TTCGTGTCCAGGATCAGATG | Forward primer for amplification of *glg55* | This paper |
| ACM100 | CTTCGACCTCTTCAATAGGC | Reverse primer for amplification of *clg55* and for Sanger Sequencing | This paper |
| PolyG-1^st^-1 | ﻿GTGACTGGAGTTCAGACGTGTGCTCTTCCGATCTGGGGGGGG GGGGGGGG | Tnseq first PCR | (1) |
| Tn5-1^st^-1 | ﻿ACCTGCAGGCATGCAAGCTTCAGGG | Tnseq first PCR | (1) |
| Tn5-2^nd^-1 | ﻿AATGATACGGCGACCACCGAGATCTACACTCTTTTCAGGGTTGAG ATGTGTATAAGAGA | Tnseq second nested PCR | (1) |
| ATTB1g-UP | CTGAACATCATCGCAGTCATCCTCATTACG | Oligo to detect pK-PIM derivative plasmid integration site | (2) |
| ATTB1g-DN | CGGCGCACGGATCGAAGTGTTC | Oligo to detect pK-PIM derivative plasmid integration site | (2) |
| ATTB2g-UP | CATAAGTAGGGATAGTTGCCAAATCTGCTC | Oligo to detect pK-PIM derivative plasmid integration site | (2) |
| ATTB2g-DN | TGTCGAGAAACGAATGCCCCAGTTTCACCC | Oligo to detect pK-PIM derivative plasmid integration site | (2) |
| H19 | ATCCAACAGGGACACCAGG | Forward oligo to linearize pCRD206 for isothermal assembly |  |
| H20 | AGTCGACCTGCAGGCATGC | Reverse oligo to linearize pCRD206 for isothermal assembly |  |
| H713 | ATAAATCCTGGTGTCCCTGTTGGATTGATCTGCGCCGTCGTCTAG | Upstream forward oligo to make *Δcgp_0844* with pCRD206 isothermal assembly homology | This paper |
| H714 | GAGGACATAAGCGCTctaTGGCTCATGCACTcatTGTGTTAAACCTCGG | Upstream reverse oligo to make *Δcgp_0844* with pCRD206 isothermal assembly homology | This paper |
| H715 | CGAGGTTTAACACAatgAGTGCATGAGCCAtagAGCGCTTATGTCCTCACTC | Downstream forward oligo to make *Δcgp_0844* with pCRD206 isothermal assembly homology | This paper |
| H716 | CAAGCTTGCATGCCTGCAGGTCGACTCGCCGCAACAACAACGCC | Downstream reverse oligo to make *Δcgp_0844* with pCRD206 isothermal assembly homology | This paper |
| H717 | GTACCGGAGGATTCCATCAAC | Forward oligo to detect and sequence *Δcgp_0844* | This paper |
| H718 | AGCAGGCTGCTGGGATTTCAG | Reverse oligo to detect and sequence *Δcgp_0844* | This paper |
| ACM293 | CCTGGTGTCCCTGTTGGATCAAGATACTTCAGGGATGTGGTG | Upstream forward oligo to make *Δcgp_0394* (*ΔppgS*) with pCRD206 isothermal assembly homology | This paper |
| ACM294 | GCACTAGGCTCTATTCCTATCATGAACGGCGTAAACATCCTGGC | Upstream reverse oligo to make *Δcgp_0394* (*ΔppgS*) with pCRD206 isothermal assembly homology | This paper |
| ACM295 | GCCAGGATGTTTACGCCGTTCATGATAGGAATAGAGCCTAGTGC | Downstream forward oligo to make *Δcgp_0394* (*ΔppgS*) with pCRD206 isothermal assembly homology | This paper |
| ACM296 | GCATGCCTGCAGGTCGACTGCGGTGGTGTCTTGGGTCTC | Downstream reverse oligo to make *Δcgp_0394* with pCRD206 isothermal assembly homology | This paper |
| ACM297 | GATCAGTGGAAACGTGGTGG | Forward oligo to detect and sequence *Δcgp_0394* (*ΔppgS*) | This paper |
| ACM298 | CTTTGCTGTCGGATTCGATG | Reverse oligo to detect and sequence *Δcgp_0394* (*ΔppgS*) | This paper |
| ACM299 | TGAGACTGTTGAATTGACTG | Forward oligo to detect and sequence *Δcgp_0658* | This paper |
| ACM300 | GGCGAGTTTTGCAGTCTAAC | Reverse oligo to detect and sequence *Δcgp_0658* | This paper |
| ACM301 | CCTGGTGTCCCTGTTGGATTTGATGGCGATCCAAGCAAG | Upstream forward oligo to make *Δcgp_0658* with pCRD206 isothermal assembly homology | This paper |
| ACM302 | CTTTGTGATTAGTTAGGGACCGTCGTCAGCATCTAGGTTCCCAC | Upstream reverse oligo to make *Δcgp_0658* with pCRD206 isothermal assembly homology | This paper |
| ACM303 | GTGGGAACCTAGATGCTGACGACGGTCCCTAACTAATCACAAAG | Downstream forward oligo to make *Δcgp_0658* with pCRD206 isothermal assembly homology | This paper |
| ACM304 | GCATGCCTGCAGGTCGACTCGGAGCATTTCTGCCATCTC | Downstream reverse oligo to make *Δcgp_0658* with pCRD206 isothermal assembly homology | This paper |
| ACM331 | CCTGGTGTCCCTGTTGGATAGCTTCGCCGAGCAAAATAG | Upstream forward oligo to make *Δcgp_0396* with pCRD206 isothermal assembly homology | This paper |
| ACM332 | GTGTTAAGCGAGCAATTTCAGGTTGCTCATCAAATATTCCTCATAC | Upstream reverse oligo to make *Δcgp_0396* with pCRD206 isothermal assembly homology | This paper |
| ACM333 | GTATGAGGAATATTTGATGAGCAACCTGAAATTGCTCGCTTAACAC | Downstream forward oligo to make *Δcgp_0396* with pCRD206 isothermal assembly homology | This paper |
| ACM334 | GCATGCCTGCAGGTCGACTGAAGGTGATGCCTTCGATGC | Downstream reverse oligo to make *Δcgp_0396* with pCRD206 isothermal assembly homology | This paper |
| ACM335 | ACTGTGTCGATGCTGAAATC | Forward oligo to detect and sequence *Δcgp_0396* | This paper |
| ACM336 | ACGTTGTTGGAGAATAGATC | Reverse oligo to detect and sequence *Δcgp_0396* | This paper |
| BH3 | tcctggtgtccctgttggatCTGCCATGATCGAGCGCG | Upstream forward oligo to make *Δcgp_2875* (*ΔprotX*) with pCRD206 isothermal assembly homology | This paper |
| BH4 | aagcggtaacAGAGGTCATTGTGATCTCTCTTTCTG | Upstream reverse oligo to make *Δcgp_2875* (*ΔprotX*) with pCRD206 isothermal assembly homology | This paper |
| BH5 | aatgacctctGTTACCGCTTCTTCCTAGTCG | Downstream forward oligo to make *Δcgp_2875* (*ΔprotX*) with pCRD206 isothermal assembly homology | This paper |
| BH6 | tgcatgcctgcaggtcgactTGGTGGCTGGGGTAAAGATTG | Downstream reverse oligo to make *Δcgp_2875* (*ΔprotX*) with pCRD206 isothermal assembly homology | This paper |
| BH9 | CAGCAAGAGTCTGGCGAAC | Forward oligo to detect and sequence *Δcgp_2875* (*ΔprotX*) | This paper |
| BH10 | CCTAGTTACTAGCGATCTCG | Reverse oligo to detect and sequence *Δcgp_2875* (*ΔprotX*) | This paper |
| BH27 | TCCTGGTGTCCCTGTTGGATATGATTGGAGGGGTTGCAAC | Upstream forward oligo to make *Δcgp_1180-cgp_1109* (Δ*porBC*) with pCRD206 isothermal assembly homology | This paper |
| BH175 | TGAAGAAGGAAAGCTTCATGATTTTTAGGGCTC | Upstream reverse oligo to make *Δcgp_1180-cgp_1109* (Δ*porBC*) with pCRD206 isothermal assembly homology | This paper |
| BH176 | CATGAAGCTTTCCTTCTTCACTGCTTAG | Downstream forward oligo to make *Δcgp_1180-cgp_1109* (Δ*porBC*) with pCRD206 isothermal assembly homology | This paper |
| BH52 | TGCATGCCTGCAGGTCGACTCGAATTCCTCGACTCTGATTC | Downstream reverse oligo to make *Δcgp_1180-cgp_1109* (Δ*porBC*) with pCRD206 isothermal assembly homology | This paper |
| BH63 | AACCAAAAGACCCAAAAAGA | Forward oligo to detect and sequence *Δcgp_1180-cgp_1109* (Δ*porBC*) | This paper |
| BH70 | TTGAAGCGATTCATGGTGGC | Reverse oligo to detect and sequence *Δcgp_1180-cgp_1109* (Δ*porBC*) | This paper |
| BH23 | TCCTGGTGTCCCTGTTGGATAGATATTGCTTTTCGACG | Upstream forward oligo to make *Δcgp_3008* (*ΔporA*) with pCRD206 isothermal assembly homology | This paper |
| BH24 | GCAGACCGATGTTTTCCATTTTAAATTCTCCTATTAAG | Upstream reverse oligo to make *Δcgp_3008* (*ΔporA*) with pCRD206 isothermal assembly homology | This paper |
| BH25 | AATGGAAAACATCGGTCTGCTTGGCTAATTAAC | Downstream forward oligo to make *Δcgp_3008* (*ΔporA*) with pCRD206 isothermal assembly homology | This paper |
| BH26 | TGCATGCCTGCAGGTCGACTGGCGGATAGCAAAACGGG | Downstream reverse oligo to make *Δcgp_3008* (*ΔporA*) with pCRD206 isothermal assembly homology | This paper |
| BH67 | CCGCTCTTCAGAGCATCC | Forward oligo to detect and sequence *Δcgp_3008* (*ΔporA*) | This paper |
| BH68 | GGTTCCATCTGGACGCAGA | Reverse oligo to detect and sequence *Δcgp_3008* (*ΔporA*) | This paper |
| BH45 | TCCTGGTGTCCCTGTTGGATGGCGAGTACGTCGACCTC | Upstream forward oligo to make *Δcgp_3009* (*ΔporH*) with pCRD206 isothermal assembly homology | This paper |
| BH46 | AGAAGTTATCAAGATCCATGAGAAATCTCCTTGAG | Upstream reverse oligo to make *Δcgp_3009* (*ΔporH*) with pCRD206 isothermal assembly homology | This paper |
| BH47 | CATGGATCTTGATAACTTCTCTTCCTAAGAG | Downstream forward oligo to make *Δcgp_3009* (*ΔporH*) with pCRD206 isothermal assembly homology | This paper |
| BH48 | TGCATGCCTGCAGGTCGACTTGAATATAGCGCTGGAAG | Downstream reverse oligo to make *Δcgp_3009* (*ΔporH*) with pCRD206 isothermal assembly homology | This paper |
| BH65 | TAACATTTCTGCAGGTCAAG | Forward oligo to detect and sequence *Δcgp_3009* (*ΔporH*) | This paper |
| BH66 | TCAGCAACTGCGCCA | Reverse oligo to detect and sequence *Δcgp_3009* (*ΔporH*) | This paper |
| BH132 | GCAGACCGATAAGATCCATGAGAAATCTCCTTGAG | Upstream reverse oligo to make *Δcgp_3008*-*3009* (*ΔporAH*) with pCRD206 isothermal assembly homology | This paper |
| BH133 | CATGGATCTTATCGGTCTGCTTGGCTAATTAAC | Downstream forward oligo to make *Δcgp_3008*-*3009* (*ΔporAH*) with pCRD206 isothermal assembly homology | This paper |
| ACM78 | CCTGGTGTCCCTGTTGGATTACATTCGCCCGCATTATCG | Upstream forward oligo to make *Δcgp_2907* (*ΔotsA*) with pCRD206 isothermal assembly homology | This paper |
| ACM79 | CGGTTCATGAGTTTTCTCCCGAGGAATCATCCATATAAGATCCG | Upstream reverse oligo to make *Δcgp_2907* (*ΔotsA*) with pCRD206 isothermal assembly homology | This paper |
| ACM80 | CGGATCTTATATGGATGATTCCTCGGGAGAAAACTCATGAACCG | Downstream forward oligo to make *Δcgp_2907* (*ΔotsA*) with pCRD206 isothermal assembly homology | This paper |
| ACM81 | GCATGCCTGCAGGTCGACTGAGTCTACGTGTGCTGCTTG | Downstream reverse oligo to make *Δcgp_2907* (*ΔotsA*) with pCRD206 isothermal assembly homology | This paper |
| ACM82 | TTTGGGCATTCAGCTTTCTG | Forward oligo to detect and sequence *Δcgp_2907* (*ΔotsA*) | This paper |
| ACM83 | ATTCCCACATTATCAACATC | Reverse oligo to detect and sequence *Δcgp_2907* (*ΔotsA*) | This paper |
| ACM224 | CCTGGTGTCCCTGTTGGATCAGCATGATGGAAATCTGAG | Upstream forward oligo to make *Δcgp_3178* (*Δpks*) with pCRD206 isothermal assembly homology | This paper |
| ACM219 | GCTGAGGCCTCAATATGGAACGGAAGAATTAGTAACGGAG | Upstream reverse oligo to make *Δcgp_3178* (*Δpks*) with pCRD206 isothermal assembly homology | This paper |
| ACM220 | CTCCGTTACTAATTCTTCCGTTCCATATTGAGGCCTCAGC | Downstream forward oligo to make *Δcgp_3178* (*Δpks*) with pCRD206 isothermal assembly homology | This paper |
| ACM225 | GCATGCCTGCAGGTCGACTTCCAGAAGAGATCCGTATCC | Downstream reverse oligo to make *Δcgp_3178* (*Δpks*) with pCRD206 isothermal assembly homology | This paper |
| ACM206 | TCGTAGCCCTGGTTAGTGTC | Forward oligo to detect and sequence *Δcgp_3178* (*Δpks*) | This paper |
| ACM207 | ACGGTCTTGCAGAAGCATCC | Reverse oligo to detect and sequence *Δcgp_3178* (*Δpks*) | This paper |
| ACM72 | CCTGGTGTCCCTGTTGGATCGCAGAAAGGTGAAACAAAC | Upstream forward oligo to make *Δcgp_0413* (*Δ*cmt1) with pCRD206 isothermal assembly homology | This paper |
| ACM73 | CGAGGGAAAGACGTATGAAGGCCTAGAAACAGATTTAACATTG | Upstream reverse oligo to make *Δcgp_0413* (*Δ*cmt1) with pCRD206 isothermal assembly homology | This paper |
| ACM74 | CAATGTTAAATCTGTTTCTAGGCCTTCATACGTCTTTCCCTCG | Downstream forward oligo to make *Δcgp_0413* (*Δ*cmt1) with pCRD206 isothermal assembly homology | This paper |
| ACM75 | GCATGCCTGCAGGTCGACTCAGTCTGAATCACATTCTTG | Downstream reverse oligo to make *Δcgp_0413* (*Δ*cmt1) with pCRD206 isothermal assembly homology | This paper |
| ACM76 | GCCACTTTCACACATAGCCG | Forward oligo to detect and sequence *Δcgp_0413* (*Δ*cmt1) | This paper |
| ACM77 | GAGAAGATGGTTCCAGAGCC | Reverse oligo to detect and sequence *Δcgp_0413* (*Δ*cmt1) | This paper |
| ACM48 | AATAAATCCTGGTGTCCCTGTTGGATCTTGGTCGATGAGCATCTCG | Upstream forward oligo to make *Δcgp_0475* (*ΔahfA*) with pCRD206 isothermal assembly homology | This paper |
| ACM269 | CCGTCGGCAAGGTGCACCAAGCACTGACCACCGAATCCGAGAACCTCTAAATGGC | Upstream reverse oligo to clone *Δcgp_0475* (*ΔahfA*) with pCRD206 isothermal assembly homology | This paper |
| ACM270 | GCCATTTAGAGGTTCTCGGATTCGGTGGTCAGTGCTTGGTGCACCTTGCCGACGG | Downstream forward oligo to clone *Δcgp_0475* (*ΔahfA*) with pCRD206 isothermal assembly homology | This paper |
| ACM51 | GCATGCCTGCAGGTCGACTTTGGCTAATGCGACGAGGTC | Downstream reverse oligo to make *Δcgp_0475* (*ΔahfA*) with pCRD206 isothermal assembly homology | This paper |
| ACM52 | TCAGCAGGCTATCGCCCACG | Forward oligo to detect and sequence *Δcgp_0475* (*ΔahfA*) | This paper |
| ACM53 | CTGATTCCAATCCAGACG | Reverse oligo to detect and sequence *Δcgp_0475* (*ΔahfA*) | This paper |
| ACM226 | CCCTGCTAAAGGAGGTAACAACAAGATGACGTGAATTCACTGGCCGTCG | Forward oligo to linearize pK-PIM derivative with homology to remove insert by isothermal assembly to create an empty vector (pACM189) | This paper |
| ACM227 | CGACGGCCAGTGAATTCACGTCATCTTGTTGTTACCTCCTTTAGCAGGG | Reverse oligo to linearize pK-PIM derivative with homology to remove insert by isothermal assembly to create an empty vector (pACM189) | This paper |
| ACM175 | CCCTGCTAAAGGAGGTAACAACAAGATGATGAAGCTTCTTCGCCGCATC | Forward oligo to amplify *cmt1* from MB001 with homology to pACM189 for isothermal assembly | This paper |
| ACM176 | CGACGGCCAGTGAATTCACGTGCCTAGGCCTCTAGCTCAAACGCAC | Reverse oligo to amplify *cmt1* from MB001 with homology to pACM189 for isothermal assembly | This paper |
| ACM178 | CTGGCATGGTCATGTCCGCAACTACTTCCC | Forward oligo to linearize pACM64 to introduce a missense mutation (GAC>GTC) to make Cmt1 catalytically dead | This paper |
| ACM177 | TGCGGACATGACCATGCCAGCAATTGCAC | Reverse oligo to linearize pACM64 to introduce a missense mutation (GAC>GTC) to make Cmt1 catalytically dead | This paper |
| BH97 | TTTTTTACCCGCACGTGAATTCACTGGCCG | Reverse oligo to amplify pACM189, removing Psod (all) and riboE1 with homology to Psod+RBS for isothermal assembly | This paper |
| BH98 | TGAGGCGCTTACTAGTTAACGTTAATTAAGAGCTCG | Forward oligo to amplify pACM189, removing Psod (all) and riboE1 with homology to Psod+RBS for isothermal assembly | This paper |
| BH99 | GTTAACTAGTAAGCGCCTCATCAGCGGT | Forward oligo to amplify Psod+RBS from chromosome with homology to pAM189 for isothermal assembly to remove riboE | This paper |
| BH100 | GAATTCACGTGGGTAAAAAATCCTTTCGTAGGTTTCC | Reverse oligo to amplify Psod+RBS from chromosome with homology to pAM189 for isothermal assembly to remove riboE | This paper |
| ACM312 | GCACGTGAATTCACTGGCCG | Forward oligo to linearize pEMH7 | This paper |
| ACM311 | GGGTAAAAAATCCTTTCGTAG  GTTTCCG | Reverse oligo to linearize pEMH7 | This paper |
| ACM290 | TACGAAAGGATTTTTTACCCATGGCAACGCGTACAGAAAAC | Forward oligo to amplify *ahfA* from MB001 with homology to pEMH7 for isothermal assembly | This paper |
| ACM180 | CGACGGCCAGTGAATTCACGTGCTTAGAGGTTCTCGGAGATCC | Reverse oligo to amplify *ahfA* from MB001 with homology to pEMH7 for isothermal assembly | This paper |

**References**

1. Lim HC, Sher JW, Rodriguez-Rivera FP, Fumeaux C, Bertozzi CR, Bernhardt TG. Identification of new components of the RipC-FtsEX cell separation pathway of Corynebacterineae. Vol. 15, PLoS Genetics. 2019. e1008284 p.

2. Oram M, Woolston JE, Jacobson AD, Holmes RK, Oram DM. Bacteriophage-based vectors for site-specific insertion of DNA in the chromosome of Corynebacteria. Gene. 2007;391:53–62.
